# Investigating the Relationship Between Cortisol, a Stress Marker, and Immune Function Across Age and Gender

**DOI:** 10.7759/cureus.85009

**Published:** 2025-05-28

**Authors:** Pradnya Padalkar, Preeti R Doshi, Meghana Padwal

**Affiliations:** 1 Biochemistry, Bharati Vidyapeeth (Deemed to Be University) Medical College, Pune, Pune, IND; 2 Pathology, Bharati Vidyapeeth (Deemed to Be University) Medical College, Pune, Pune, IND

**Keywords:** associated factors, cortisol, immune function, stress, stress marker

## Abstract

This study investigates the relationship between cortisol, a well-known stress marker, and immune function, focusing on how cortisol levels affect immune responses across different age groups and genders. Stress can be acute or chronic, both of which influence physiological processes in the body. Cortisol plays a key role in this process, with acute stress leading to brief elevations of cortisol that can enhance immune function, while chronic stress leads to dysregulation and immune suppression. This retrospective study, conducted at a tertiary care hospital, analyzed data from patients who underwent cortisol and complete blood count (CBC) tests between April and September 2024. The data, collected from both male and female participants of various age groups, revealed significant correlations between cortisol levels and immune parameters like total leukocyte count (TLC), lymphocyte percentage, and neutrophil percentage. Notably, cortisol was positively correlated with TLC, while it showed a weak negative correlation with lymphocyte percentage, suggesting a potential reduction in immune responsiveness with elevated cortisol levels. Gender differences were also observed, with females exhibiting a stronger negative correlation between age and lymphocyte percentage, while males showed significant correlations between cortisol levels and immune markers. These findings underscore the complex interplay between stress, cortisol, and immune function, providing insights into stress-related health conditions such as infections and autoimmune disorders.

## Introduction

Stress is a physiological response to internal and external stimuli that challenge the ability of body to maintain homeostasis. Stress can be manifested in two primary categories: acute stress and chronic stress. Acute stress is typically short-lived, ranging from a few minutes to a few hours; on the other hand, chronic stress persists over an extended period, ranging from several hours to weeks or even months [1,2]. Both forms of stress have been studied extensively for their impact on multiple physiological processes, mainly their influence on the immune system [2].

The key biological mediator of stress is cortisol, which is a glucocorticoid hormone synthesized and released by the adrenal glands in response to stressors. The cortisol hormone plays a central role in the body’s “fight or flight” response by mobilizing energy, regulating metabolism, and modulatory inflammation [3,4]. Under normal physiological conditions, cortisol is crucial in order to maintain homeostasis. However, dysregulation in the cortisol level due to prolonged or excessive stress can have significant consequences on the multiple organ systems, including the immune system [5].

The immune system of the body serves as the primary defense mechanism against infections, diseases, and other harmful agents. The emerging research highlights a complex and dynamic interplay between immune function and stress, with cortisol acting as a key regulator in the interaction [3,6]. The increased level of cortisol has been shown to inhibit immune function by inhibiting the production of cytokines and impairing T-cell activity, thereby increasing susceptibility to infections and other immune-linked disorders [4,7]. On the other hand, a transient elevation in the cortisol level during acute stress may enhance a certain immune response, suggesting the duration and the magnitude of cortisol exposure are critical factors in determining the immune outcomes [8].

Examining the cause-and-effect relationship of stress and immune function, this research investigates the possible impact of cortisol activity as a stress marker and neuroregulation of immune functions. It analyzes variations in the cortisol levels and their subsequent effects on the immune function to elucidate the broader implications of stress-induced immune alterations. Certain considerations must be addressed in order to assess the future health implications, such as susceptibility to infections, the occurrence of autoimmunity, and other diseases influenced by stress conditions [9]. This retrospective study will help determine the precise physiological mechanisms underlying stress, cortisol, and immune system interaction while also providing insight into stress-induced health discomfort levels. Thus, the aim of the study is to check the association of cortisol level, stress marker, and immune function.

## Materials and methods

A retrospective study was conducted over a six-month period from April to September 2024 in the Department of Biochemistry and Pathology at a tertiary care hospital and laboratory. The study population consisted of patients visiting to tertiary care hospital and were asked for investigations like cortisol and complete blood count (CBC). Every participant gave their written informed consent. There was tight adherence to the inclusion and exclusion criteria. The inclusion criteria were participants of both genders undergoing investigations for serum cortisol and CBC, and the exclusion criteria were known cases of immune diseases and/or a history of consumption of antioxidant drugs. Prior to beginning the actual study, the institutional ethics committee's (IEC) consent was obtained, named Bharati Vidyapeeth (Deemed to Be University) Medical College (IEC no.: BVDUMC/IEC/163, dated 13.04.2024). All the data of participants were obtained from a tertiary care hospital and the hospital information system (HIS) for the haematological and biochemical investigations. CBC, including white blood cell count and differential counts, was performed using the Coulter principle on the DxH 800 analyser (Beckman Coulter, Brea, CA, USA). Serum cortisol levels were measured using chemiluminescent microparticle immunoassay (CMIA) on the Alinity ci Integrated Platform - Immunoassay module (Abbott Laboratories, Abbott Park, IL, USA).

Statistical analysis

All statistical analyses were conducted by IBM SPSS Statistics for Windows, Version 29 (Released 2021; IBM Corp., Armonk, New York, United States), while Microsoft Excel 2010 (Microsoft® Corp., Redmond, WA, USA) was used for graphical representation purposes. To ensure that the data is reliable, each record was double-checked for potential errors while inputting. Quantitative data was analyzed using descriptive statistics, whereas categorical data was graphically represented using percentages and frequencies. Diagrams were also included where required to provide a better picture of the data. To ensure the trustworthiness of the findings, all data to be analyzed was thoroughly checked for completeness and accuracy. The kind of data and the study aims influenced the use of statistical tests. To assess the differences in cortisol levels between stress markers and immune function using the CBC tests, the independent t-test was carried out. Besides, the correlation between stress markers and immune function was discovered through Pearson's correlation coefficient test. A p-value less than 0.05 (p < 0.05) was deemed to be statistically significant, indicating the existence of meaningful associations or differences in the data.

## Results

In the correlation analysis, the association between age, cortisol levels, and immune markers (total leukocyte count (TLC), lymphocytes, and neutrophils) in different age groups is studied (<20, 20-60, >60 years). The research suggests that the influence of cortisol levels (as an indicator of stress) on immune functioning is different and is age-specific (Table 1).

**Table 1:** Correlation Analysis of Age, Cortisol, and Immune Markers Across Different Age Groups TLC: total leukocyte count

Age Category	Variables	Age	Cortisol (Morning/Random) (nmol/L)	TLC (4000-11000 cu mm)	Lymphocyte (20-40%)	Neutrophil (40-80%)
<20 Years	Age	1	0.032	-0.577	-0.121	0.047
	Cortisol	0.032	1	0.865	0.239	-0.294
	TLC	-0.577	0.865	1	-0.274	0.364
	Lymphocyte	-0.121	0.239	-0.274	1	-0.991
	Neutrophil	0.047	-0.294	0.364	-0.991	1
>60 Years	Age	1	-0.225	-0.223	-0.259	0.298
	Cortisol	-0.225	1	0.859	-0.333	0.171
	TLC	-0.223	0.859	1	-0.391	0.219
	Lymphocyte	-0.259	-0.333	-0.391	1	-0.875
	Neutrophil	0.298	0.171	0.219	-0.875	1
20-60 Years	Age	1	-0.08	0.595	-0.713	0.69
	Cortisol	-0.08	1	0.057	-0.053	0.091
	TLC	0.595	0.057	1	-0.623	0.611
	Lymphocyte	-0.713	-0.053	-0.623	1	-0.964
	Neutrophil	0.69	0.091	0.611	-0.964	1

Cortisol and TLC have a strong positive correlation, particularly in young patients, possibly due to increased inflammation through immune system cells (r = 0.865, p < 0.05). The strong negative link between lymphocytes and neutrophils (r = -0.991, p < 0.01) suggests a well-immunized state, where a positive balance of one subset leads to a decrease in the other.

Like in the younger group, among those 60 and above, the cortisol content still shows a strong positive correlation with TLC (r = 0.859, p < 0.01). Nevertheless, the relationship with lymphocytes as well as neutrophils gets weaker, which implies that the stress hormones have reduced the immune system's regulating ability in elderly people. Though the person is getting older, the constant negative link between lymphocytes and neutrophils (r = -0.875, p < 0.01) ensures that the immune balance stays the same.

For the age group of 20 to 60, there is a very positive relationship between age and TLC (r = 0.595, p < 0.01) that indicates a gradual increase in leukocyte count with aging. Another significant factor in the response to aging has been the negative correlation of age with lymphocytes (r = -0.713, p < 0.01), but it is related to a positive relationship with neutrophils (r = 0.690, p < 0.01), which means the ratio of these cell types changes towards a neutrophil-dominant immune response, and perhaps the latter is an age-associated immune adaptive process.

Table 2 depicts a variety of correlation coefficients associated with age, cortisol levels, TLC, lymphocyte percentage, and neutrophil percentage, which are stratified by gender. Pearson correlation values, in addition to their statistical significance (p-values), are offered.

**Table 2:** Correlation Analysis of Age, Cortisol, and Immune Parameters (TLC, Lymphocytes, and Neutrophils) by Gender TLC: total leukocyte count

Gender	Variables	Age (years)	Cortisol (Morning/Random) (nmol/L)	TLC (cu mm)	Lymphocyte (%)	Neutrophil (%)
Female	Age	1	0.219	0.385	-0.691	0.666
	Sig. (2-tailed)	NA	0.115	0.006	0	0
	N	66	53	49	49	49
	Cortisol	0.219	1	0.536	-0.166	0.149
	Sig. (2-tailed)	0.115	NA	0	0.275	0.329
	N	53	53	45	45	45
	TLC	0.385	0.536	1	-0.509	0.508
	Sig. (2-tailed)	0.006	0		0	0
	N	49	45	49	49	49
	Lymphocyte	-0.691	-0.166	-0.509	1	-0.98
	Sig. (2-tailed)	0	0.275	0	NA	0
	N	49	45	49	49	49
	Neutrophil	0.666	0.149	0.508	-0.98	1
	Sig. (2-tailed)	0	0.329	0	0	
	N	49	45	49	49	49
Male	Age	1	0.133	0.383	-0.744	0.73
	Sig. (2-tailed)	NA	0.358	0.012	0	0
	N	55	50	42	43	43
	Cortisol	0.133	1	0.452	-0.236	0.236
	Sig. (2-tailed)	0.358	NA	0.004	0.142	0.143
	N	50	50	39	40	40
	TLC	0.383	0.452	1	-0.6	0.564
	Sig. (2-tailed)	0.012	0.004	NA	0	0
	N	42	39	42	42	41
	Lymphocyte	-0.744	-0.236	-0.6	1	-0.954
	Sig. (2-tailed)	0	0.142	0	NA	0
	N	43	40	42	43	42
	Neutrophil	0.73	0.236	0.564	-0.954	1
	Sig. (2-tailed)	0	0.143	0	0	NA
	N	43	40	41	42	43

Female group

The study found that age is highly negatively correlated to the level of lymphocytes (-0.691, p < 0.001) in women, suggesting that lymphocyte levels decrease with age. Analogous to that, age was positively correlated with neutrophil percentage (0.666, p < 0.001), which implies that an increase in neutrophil level due to aging occurred. Moreover, there was a moderate positive correlation between TLC and age (0.385, p = 0.006).

Cortisol levels showed a strong positive correlation with TLC (0.536, p < 0.001), but they did not significantly correlate with lymphocyte (-0.166, p = 0.275) or neutrophil levels (0.149, p = 0.329). This implies that although cortisol is associated with a general increase in leukocytes, its effect on certain immune cell types is less direct.

A significant inverse correlation between TLC and lymphocytes (-0.509, p < 0.001) and a positive correlation with neutrophils (0.508, p < 0.001) have been demonstrated. Lymphocyte and neutrophil percentages had a very high negative correlation (-0.980, p < 0.001), showing the expected inverse correlation where an increase in one is associated with a decrease in the other.

Male group

A relationship that exists between them is shown by the fact that among males, lymphocyte percentage had a substantially lower correlation with age (-0.744, p < 0.001) and a substantially stronger correlation with neutrophils (0.730, p < 0.001), a higher age being somewhat very like the situation with women. In addition, the correlation of age with TLC was slightly positive (0.383, p = 0.012).

In males, high cortisol levels were associated with more TLC (0.452, p = 0.004), which was the same situation for females. However, cortisol had no direct correlation with lymphocyte (-0.236, p = 0.142) or neutrophil levels (0.236, p = 0.143), suggesting that cortisol mainly affects overall leukocyte levels instead of specific immune subsets.

TLC had a significant negative correlation with lymphocytes (-0.600, p < 0.001) and a positive correlation with neutrophils (0.564, p < 0.001). The percentage of lymphocytes and neutrophils once more demonstrated a strong inverse relationship (-0.954, p < 0.001), a condition that was almost the same as the female group.

In Table 3, the correlations between cortisol (morning/random), TLC, lymphocyte percentage, and neutrophil percentage were analyzed. The direction and strength of the associations shown by Pearson’s correlation coefficient are highlighted in this part. P-values show the statistical significance of each correlation, with a value less than 0.05 showing a significant association.

**Table 3:** Correlation Analysis of Cortisol, TLC, Lymphocytes, and Neutrophils TLC: total leukocyte count

Variables	Cortisol (Morning/Random) (nmol/L)	TLC (4000-11000 cu mm)	Lymphocyte (20-40%)	Neutrophil (40-80%)
Cortisol (Morning/Random)	1	0.498	-0.216	0.197
p-value	-	0	0.04	0.064
N	108	90	90	89
TLC (4000-11000 cumm)	0.498	1	-0.521	0.512
p-value	0	-	0	0
N	90	93	93	92
Lymphocyte (20-40%)	-0.216	-0.521	1	-0.966
p-value	0.04	0	-	0
N	90	93	93	92
Neutrophil (40-80%)	0.197	0.512	-0.966	1
p-value	0.064	0	0	-
N	89	92	92	92

The findings demonstrate a moderate positive correlation (r = 0.498, p < 0.01) between cortisol and TLC, which signifies that higher cortisol levels are associated with a higher TLC. On the other hand, a weak but significant negative correlation (r = -0.216, p = 0.04) is noticed between cortisol and lymphocyte percentage, indicating that the elevated cortisol levels may be associated with a small amount of reduction in the size of the body's white blood cells. Nevertheless, the correlation between cortisol and neutrophils (r = 0.197, p = 0.064) is insignificant, stating that cortisol may have a small tendency to increase neutrophil levels, but this effect is not supported by strong statistical evidence.

An investigation of the relationship between TLC and other immune parameters discovered a strong negative correlation (r = -0.521, p < 0.01) between TLC and lymphocytes, which means that with the rise of the TLC, the percentage of lymphocytes drops significantly. On the contrary, the strong positive correlation (r = 0.512, p < 0.01) demonstrates a close connection between TLC and neutrophils, i.e., an increase in TLC is related to a higher level of neutrophils.

Table 3 reveals a strong negative correlation between lymphocytes and neutrophils (r = -0.966, p < 0.01). This discovery demonstrated that the lymphocyte-neutrophil balance, which has long been known, persists, as does a decrease in the percentage of neutrophils, which occurs when the percentage of lymphocytes increases.

## Discussion

Stress responses can profoundly affect the digestive system. During stressful periods, digestion is suppressed; conversely, once the stress diminishes, digestive activity typically escalates. This fluctuation may adversely impact digestive health and could potentially result in the formation of ulcers. Moreover, adrenaline released during a stress response may also facilitate the development of ulcers. Furthermore, stress responses impose extra pressure on the circulatory system, resulting in an elevated heart rate and other associated effects. Stress may undermine the immune system by elevating blood pressure.

Furthermore, stress responses impose further stress on the circulatory system, resulting in an elevated heart rate and other associated effects. Stress may undermine the immune system by elevating blood pressure.

Higher heart rates contribute to a faster accumulation of cholesterol on arterial walls. Additionally, hypertension can cause small lesions in these walls, creating sites where cholesterol often becomes trapped. Moreover, stress can indirectly lead to health problems by fostering detrimental coping mechanisms, such as smoking [10].

The significant discoveries contributed to the expanding corpus of knowledge that underscores particular brain circuits as integral components of the immune system. The notion of the brain acting as a functional component of the immune system is not novel; however, elucidating the specific connections and signalling mechanisms that influence different facets of the immune response presents new and focused opportunities for intervention. These findings offer essential insights into the function of corticotropin-releasing hormone-paraventricular nucleus (CRH-PVN) neurons in specific immune response mechanisms. Besides employing neuroendocrine signals to modulate immune cell movement, CRH-PVN neurons extend projections to the splenic nerve, promoting the generation of plasma cells in a T cell-dependent immune response [8].

In individuals under 20 years of age, elevated cortisol, commonly linked to stress, may be correlated with increased total lymphocyte count (TLC). This relationship may indicate the physiological function of cortisol in regulating immune responses, especially during stress or inflammation in younger individuals. An inverse relationship is anticipated, as neutrophil predominance typically arises during acute inflammation or infection, while lymphocytes are comparatively diminished in these circumstances. The age and other variables in this cohort suggest that, within this limited age range, age may not significantly influence cortisol or immune parameters.

The findings indicate that psychological stress may generally impact the immune system, resulting in immune suppression and an imbalance that could potentially incite an autoimmune response against B cells. Moreover, children subjected to psychological stress, especially from traumatic events, exhibit an immune response that targets autoantigens associated with type 2 diabetes mellitus [9].

In the older age group (>60 years), the findings are analogous to those of the younger group. This relationship may indicate an age-related stress response or chronic inflammation, prevalent in older individuals due to immune senescence or underlying health conditions. The elevation of cortisol may promote leukocyte proliferation as a component of the body's stress response. A correlation was identified between lymphocyte percentage and neutrophil percentage, aligning with anticipated immunological dynamics in which neutrophils predominate during inflammatory conditions. Notably, age did not exhibit a significant correlation with cortisol or immune parameters. This indicates that in this older demographic, factors such as chronic illness, stress, or pharmacological treatments may exert a greater impact on cortisol and leukocyte dynamics than age alone.

In the middle-aged cohort (ages 20-60), the correlation between cortisol levels and TLC confirms previous findings that cortisol positively affects leukocyte count. This correlation may be associated with acute or chronic stress, which is common in this demographic due to occupational and lifestyle influences. The percentage of lymphocytes and neutrophils shows that these two immune parameters are linked, which is in line with how they work biologically in stress and immune responses. Cortisol levels and age exhibited no significant correlation, suggesting that age is not a primary factor influencing cortisol levels or immune parameters in this cohort.

These findings align with the impact of cortisol on immune parameters. In line with previous research, our results show that high cortisol levels cause neutrophils to move around and lymphocytes to decrease in number. The literature has documented age-related differences in immune function and cortisol responses, corroborating our findings.

Gender-specific associations

In both male and female participants, age exhibited a consistent correlation pattern with immune markers. Our findings revealed a gender disparity in immune response, as detailed in Table 2. Men exhibited significantly (p < 0.05) elevated neutrophil and leukocyte values compared to women, suggesting that age-related immune alterations are more pronounced in men.

The gender-based correlations indicated that the relationship between stress and lymphocyte and neutrophil levels was not significant for either men or women (Table 2). The correlation between stress and TLC activity was significant for both genders.

The results indicate notable differences in immunological and hormonal responses between sexes. Males exhibited marginally more pronounced age-related alterations, aligning with research indicating that immune aging patterns are gender specific. Cortisol similarly influenced TLC in both genders; however, its impact on immune cell distribution remains unclear and requires further investigation [6].

According to a number of studies, women are twice as likely as men to experience stress or depression in their lifetime. This could be because of a variety of factors, such as hormonal differences, cultural backgrounds, gender differences in social activities, and how people react to stressors [11-13].

The onset of perimenopause in women may be a contributing factor to the increase in cortisol levels associated with aging. Substantial life alterations during this period may be associated with sexual activity, endocrine metabolism, and familial conflicts that lead to anxiety and depression [6,14].

Cortisol is essential in regulating stress responses, hence, it is commonly termed the "stress hormone." It impacts the body in various ways, including mobilizing energy reserves through enhanced glucose production, inhibiting non-essential processes such as reproduction and digestion, modifying immune responses, and affecting mood and memory.

This study's results elucidate the correlation between cortisol levels and immune parameters, namely TLC, lymphocyte percentage, and neutrophil percentage. These findings are especially pertinent in comprehending how stress, indicated by cortisol levels, affects immune function.

Cortisol and TLC

A moderate positive correlation aligns with the established physiological effects of cortisol, a glucocorticoid hormone secreted in response to stress. Cortisol is recognized for its role in mobilizing leukocytes, especially neutrophils, into the bloodstream during the body's acute stress response [15].

Cortisol and lymphocyte percentage

The link is statistically significant despite its weakness. The well-established immunosuppressive effects of cortisol on lymphocytes are supported by this finding. By lowering lymphocyte numbers, prolonged stress and high cortisol levels may impair the adaptive immune response [16].

Cortisol and neutrophil percentage

No statistically significant association was seen between cortisol levels and the proportion of neutrophils. Although the connection was positive, the absence of significance suggests that other variables may affect neutrophil counts, including acute infections, inflammation, or individual variability. This result indicates that cortisol's influence on neutrophils may not be as direct or significant in the present group. Subsequent research with bigger sample numbers or more controlled environments may elucidate this association

Prolonged exposure to increased cortisol levels is associated with immunosuppression, mostly due to diminished lymphocyte function, potentially hindering the body's capacity to fight infections. The absence of a substantial link with neutrophils may indicate individual variances in stress response or differing baseline inflammation levels, both of which are recognized to affect neutrophil dynamics. The poor connection with lymphocytes indicates that variables other than cortisol may influence immunological responses.

Clinical research has shown that psychological stress activates innate immune responses in humans; for instance, medical interns exhibited increased stress intensity and frequency when working in the intensive care unit (ICU) compared to their off-duty periods. The increase in circulating neutrophils and monocytes during ICU work illustrates the correlation between leukocyte mobilization and psychological stress. Elevated blood monocytes are associated with poor socioeconomic position, perhaps resulting in chronic social stress. This paper presents an overview of the impact of stress on the brain and peripheral immune systems. Research indicates that stress-induced immunological responses significantly influence depressed and anxiety-like behaviors; however, these responses are intricate and contingent upon host circumstances and stressors. Stress activates both innate and adaptive immune cells in the periphery via the coordinated activities of sympathetic nerves and glucocorticoids, as well as the effect of gut microbiota. Neutrophils and monocytes exit the bone marrow in reaction to stress [7].

Health and psychological factors, including strained relationships, competitive or adverse social interactions, and loneliness, have been associated with immune function and stress. These mediators have also been associated with heightened pro-inflammatory responses to stress. The significance of other mediators, such as adequate sleep, in the stress-immunity link is increasingly recognized. Recent studies indicate that a single night of total sleep deprivation significantly elevates neutrophil numbers while diminishing neutrophil functionality in healthy males. This approach may ultimately result in the creation of behavioural or pharmacological interventions, especially aimed at persons most susceptible to negative health consequences [4].

Research indicates that whereas chronic stress exposure may yield more detrimental consequences, acute stress exposure enhances both innate and acquired immunity, hence improving an organism's likelihood of surviving possible injuries and pathogen invasion [14].

Stress management strategies are crucial for improving overall wellness as they mitigate the adverse effects of chronic stress on the immune system. To enhance resistance against the adverse effects of chronic stress, we may explore several stress management measures to bolster immune function. Such as lifestyle modifications, mindfulness practices like yoga and meditation, and social support and relationships, among others.

## Conclusions

The present study investigated the relationships between age, cortisol levels, TLC, lymphocyte percentage, and neutrophil percentage, stratified by gender. The findings revealed significant patterns of correlation, highlighting potential links between immune function, stress hormones, and demographic factors. This study highlights significant correlations between age, cortisol levels, and immune parameters, with notable gender-specific patterns. These findings contribute to our understanding of immune regulation in the context of aging and stress, paving the way for future research to explore underlying mechanisms and clinical applications.

The findings further contribute to a better understanding of how stress impacts immune function and emphasize the importance of managing stress to maintain immune balance. Future research should consider longitudinal studies and control for potential confounding factors to provide a more comprehensive understanding.

## References

[1] Dhabhar FS (2014). Effects of stress on immune function: the good, the bad, and the beautiful. Immunol Res.

[2] Bae YS, Shin EC, Bae YS, Van Eden W (2019). Editorial: stress and immunity. Front Immunol.

[3] Yosef N, Regev A (2016). Writ large: genomic dissection of the effect of cellular environment on immune response. Science.

[4] Morey JN, Boggero IA, Scott AB, Segerstrom SC (2015). Current Directions in stress and human immune function. Curr Opin Psychol.

[5] Herbert TB, Cohen S (1993). Stress and immunity in humans: a meta-analytic review. Psychosom Med.

[6] Cañas-González B, Fernández-Nistal A, Ramírez JM, Martínez-Fernández V (2020). Influence of stress and depression on the immune system in patients evaluated in an anti-aging unit. Front Psychol.

[7] Ishikawa Y, Furuyashiki T (2022). The impact of stress on immune systems and its relevance to mental illness. Neurosci Res.

[8] Bains JS, Sharkey KA (2022). Stress and immunity - the circuit makes the difference. Nat Immunol.

[9] Carlsson E, Frostell A, Ludvigsson J, Faresjö M (2014). Psychological stress in children may alter the immune response. J Immunol.

[10] Dorian B, Garfinkel PE (1987). Stress, immunity and illness - a review. Psychol Med.

[11] Wharton W, Gleason CE, Miller VM, Asthana S, Neal-Perry G (2012). Hormonal factors associated with depression in women. Front Neuroendocrinol.

[12] Altemus M, Sarvaiya N, Epperson CN (2014). Sex differences in anxiety and depression: clinical perspectives. Front Neuroendocrinol.

[13] Kuehner C (2017). Why is depression more common among women than among men?. Lancet Psychiatry.

[14] Ravi M, Miller AH, Michopoulos V (2021). The immunology of stress and the impact of inflammation on the brain and behavior. BJPsych Adv.

[15] Swain S, Dalai N, Mohapatra S, Mishra SR, Mahapatra APK, Kundu AK, Pamia J (2023). Effect of heat stress on haemato-biochemical and corticosterone level of coloured synthetic broiler male line. Pharma Innovation J.

[16] Dong T, Zhi L, Bhayana B, Wu MX (2016). Cortisol-induced immune suppression by a blockade of lymphocyte egress in traumatic brain injury. J Neuroinflammation.

